# Pharmacological Activity of Eriodictyol: The Major Natural Polyphenolic Flavanone

**DOI:** 10.1155/2020/6681352

**Published:** 2020-12-12

**Authors:** Zirong Deng, Sabba Hassan, Muhammad Rafiq, Hongshui Li, Yang He, Yi Cai, Xi Kang, Zhaoguo Liu, Tingdong Yan

**Affiliations:** ^1^School of Pharmacy, Nantong University, 19 Qixiu Road, Nantong, Jiangsu 226001, China; ^2^Department of Physiology and Biochemistry, Cholistan University of Veterinary and Animal Sciences (CUVAS), Bahawalpur 63100, Pakistan; ^3^The Second People Hospital of Dezhou, Dezhou 253022, China; ^4^BayRay Innovation Center, Shenzhen Bay Laboratory, Shenzhen 518132, China; ^5^Center of Pharmaceutical Research and Development, Guangzhou Medical University, Guangzhou, Guangdong 511436, China; ^6^Center of Translational Medicine, Chemical Biology Institute, Shenzhen Bay Laboratory, Shenzhen 518132, China

## Abstract

Eriodictyol is a flavonoid that belongs to a subclass of flavanones and is widespread in citrus fruits, vegetables, and medicinally important plants. Eriodictyol has been anticipated to explain the method of its activity via multiple cellular signaling cascades. Eriodictyol is an effective natural drug source to maintain higher health standards due to its excellent therapeutic roles in neuroprotection, cardioprotective activity, hepatoprotective activity, antidiabetes and obesity, and skin protection and having highly analgesic, antioxidant, and anti-inflammatory effects, antipyretic and antinociceptive actions, antitumor activity, and much more. This review aims to highlight the modes of action of eriodictyol against various diseases via multiple cellular signaling pathways.

## 1. Introduction

**Figure 1:**
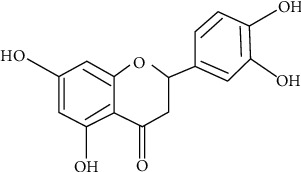
The chemical structure of eriodictyol.

The flavonoid eriodictyol (ER) [(S)-2-(3,4-dihydroxyphenyl)-5,7-dihydroxy-2,3-dihydrochromen-4-one] (Figure 1) belongs to flavanones subclass [1] mostly found in citrus fruits, vegetables, and most of the medicinal plants [2]. Flavonoids are the polyphenols essentially present in human diet on regular basis and mainly occur in natural herbs [3]. Phytomedicine is an essential part of the traditional Chinese medication system which basically works by scavenging free radical and possesses anti-inflammatory effects. It is assumed that phytochemicals have a noteworthy part in human healthcare systems and account for over 40% of professionally prescribed medicines that are essentially homegrown [4]. Therapeutically, phytomedicine has plenty of advantages over synthetic drugs in terms of low toxicity with reduced or none of the side effects [5]. Phytomedicine is an important source for the exploration of new drugs that help cure various health issues and diseases [6].

ER potentially drives a huge number of cellular signaling pathways to cure different diseases due to its numerous therapeutic effects reported for different ailments. ER is a vital part of dietary supplements and being a part of food poses extraordinary antioxidant effects for reducing the risks of any health issues [7].

## 2. Sources of Eriodictyol (ER)

ER is a flavanone mainly extracted from yerba Santa Clause (*Eriodictyon californicum*), a plant local to North America [8]. ER is one of the four flavanones isolated from this plant as having taste-altering properties, the other three being sterubin, homoeriodictyol, and its sodium salt [9]. ER is likewise found in *Eupatorium arnottianum* [10], its glycosides (eriocitrin) in lemons and *Rosa canina* [11], and in the twigs of *Millettia duchesnei* [9]. ER has also been reported to be extracted from the stem bark of *Piptadeniastrum africanum* plant, which is widely used in African traditional remedies [12].

## 3. Pharmacological Properties

A vast majority of flavonoids present in plants are complexed with carbohydrates, e.g., *β*-glycosides. The flavonoid ER generally retained as such that gradually expended in the digestive tract as glucuronidation following the action of gut microbiota and is metabolized through methoxylation in the liver and turned into restructured homoeriodictyol. Formed metabolites are in the long run used in the kidneys, delivering glucuronic acids having corrosive or potential sulfate groups from them and enhancing their biliary and urinary discharge. ER as a formed structure has also been identified in blood plasma and urine samples at 4 h and 24 h, respectively [13]. Moreover, ER has a broad spectrum of pharmacological activities, and it was recently reported that its roles in curing various diseases has drawn much attention for this compound.

### 3.1. Protection in Cardiovascular Issues

Coronary illness is created because of excessive accumulation of oxidative stress generated by various reactive oxygen species (ROS), and low-density lipoprotein (LDL) cholesterol. Nonetheless, it is also demonstrated that ER reduces the chances of myocardial ischemia/reperfusion (I/R) injury in the SD rodent by stifling support of incendiary and myocardial apoptosis responses by modulating Janus kinase 2 (JAK2) pathway [14]. In continuation to this, ER treatment helps improve the cardiomyocyte injury mainly by the activation of the B cell lymphoma-2 (Bcl-2) and Bcl-2-related factor X (BAX) signaling pathway via caspase-3 signaling pathway [15]. Moreover, ER diminishes the activation of C-responsive protein, p38, and JNK2 articulation, which leads to a decrease in NO accumulation and a decrease in expression of VEGF and endothelial bond factor articulation eventually reduces the scar formation and vascular stenosis gives rise to atherosclerosis [16]. In addition, ER triggers ERK/Nrf2/ARE intervened upregulation of heme oxygenase (HO-1) expression in human endothelial cells which is legitimately linked with vascular stability against oxidative stress mediated endothelial injury. This recommends that ER-mediated regulation of HO-1 expression is a promising tool for combating cardiovascular ailments [13].

### 3.2. Skin Protection

Skin is the largest organ of the human body which is constantly exposed to external environment. Atopic dermatitis is chronic but inflammatory dermatological condition having some allergic signs as well. ER restrains immunoglobulin E (IgE)/Ag-induced type I hypersensitivity mainly via reducing the expression of certain inflammatory interleukin-4 (IL-4) along with ceramide kinase [17]. The extracts of *Aspalathus linearis* (Rooibos) triggers muscarinic M3 receptor to increase the dryness in the skins, as dryness may influence life style and pleasure.

Moreover, eriodictyol-6-C-*β*-D-glucoside (E6CG) dynamically influences the secretions from exocrine organs. The pharmacokinetics of the dissemination of E6CG in exocrine organs have not been fully elucidated in the mice exposed to rooibos extracts. LC–MS/MS was implied to distinguish E6CG without any change in the structural change and applied on C57BL/6 mice that showed rooibos extracts helping in moving E6CG into the blood plasma shortly after the exposure. Huge amounts of E6CG are present in the submandibular, sublingual, parotid, and lacrimal organs and in the perspiration organs in palm skin. This reports the mechanism of rooibos mediated separation of E6CG in the skin may be utilized in medicated personal care products for the nourishment and improving dryness of the skin [18].

### 3.3. Dryness of Mouth, Eye, and Skin

Various foods containing rooibos extracts rich in E6CG have significantly reduced dryness of human oral cavity (*P*=0.019), eyes (*P*=0.006), and skin (*P*=0.030) after about fourteen days of application at a 100 mg concentration (0.21 mg E6CG) of crude rooibos dosage. The clinical examinations showed that continuous consumption of rooibos extracts significantly removed dryness of mouth, eye, and skin [19].

### 3.4. Antitumor Activity

The uncontrolled growth of the cells leads to the formation of tumors. Flavonoids are involved in various cellular pathways for lowering malignant growths of tumors. The anticancer potential of flavonoids may incur at multiple steps via the hindrance in cell development, multiplication, and metastases even [20]. A recent study demonstrated that ER applies its anticancer effects by inducing apoptosis, cell cycle arrest at G2/M phase through restraining the mTOR/PI3K/Akt cascade [21], showing the involvement of ER through some planned effect especially in the treatment of lung carcinoma. Additionally, ER represses lipid peroxidation and a modulatory effect on preneoplastic injuries in colon by its cytoprotective potential [22].

The downregulation of PI3K/Akt signaling has a basic role in cell proliferation, survival, and autophagy [23]. The increased downregulation in PI3K/Akt expression leads to reduced tumorigenesis [24]. The upregulation in the expression of PI3K pathway may lead to enhanced malignancy by increasing cell proliferation and make the tumor cell resistant by avoiding apoptosis [25]. The NF-*κ*B signaling pathway is of extreme importance that directs different cellular cascades activation to induce disease advancement and tumorigenesis [26]. Upregulation of the NF-*κ*B expression promptly leads to its movement from cytoplasm into the nucleus which may advance malignant growth metastasis [27]. In this regard, focusing on modulation of the PI3K/Akt/NF-*κ*B signaling pathway may be a promising approach while treating the malignant growth. ER could efficiently affect insulin-mediated glucose take-up in human hepatocellular carcinoma cells by controlling the PI3K/Akt signaling pathway [28].

Moreover, ER was found to diminish nitric oxide (NO), TNF-*α*, IL-6, and IL-1*β* creation in LPS-activated RAW264.7 cells through downregulation of the MAPK/NF-*κ*B signaling pathways [29]. Herein, the manuscript illustrates that the reducing effects of ER on the glioma may be the suppression of the PI3K/Akt/NF-*κ*B pathway. The study shows that ER may partly induce apoptosis by repressing the phosphorylation of PI3K, Akt, and NF-*κ*B. Moreover, ER inactivates PI3K/Akt/NF-*κ*B pathway, upregulates Bax, and cleaves PARP expression. The involvement of ER in the pro-apoptotic pathways suggests that the former actively triggers apoptosis in U87MG and CHG-5 cells mainly by suppressing the PI3K/Akt/NF-*κ*B pathway [30].

### 3.5. Effect on Central Nervous System (CNS)

Stroke is an unpredictable problem that happens due to excessive oxidative pressure associated pathways in its pathogenesis. The nuclear factor erythroid-2-related factor 2/antioxidant response elements (Nrf2/ARE) pathway is an important signaling mechanism involved in activating phase-II detoxification enzymes along with cytoprotective proteins taking part in neuroprotection during stroke. Being a novel activator of Nrf2, eriodictyol-7-O-glucoside (E7G) ensures protection for cerebral ischemic injury through its Nrf2/ARE pathway in neuroprotection. In cultured astrocytes, E7G enhances the nuclear translocation of Nrf2 and triggers the activation of downstream Nrf2/ARE genes and provides antioxidant responses. These neuroprotection effects are shown by the activation of Nrf2/ARE antioxidant pathway triggered by E7G which is linked with direct reduction of oxidative pressure prompted ischemic injury and might be a promising way out for successful intercession in stroke [31].

### 3.6. Antioxidant Activity

The potential of scavenging the free ROS or RNA is termed as the antioxidant potential of any compound. The extracts rich in flavonoid can chelate metal particles and free radicals and associate with the metabolic products to provide a vital protection against various cellular degradation processes [32]. A recent study unleashed how ER diminishes lipid peroxidation in isoproterenol-induced myocardial infarcted rodents after 45 days of exposure to the drug [33]. ER provides increased cell protection by enhanced antioxidant activity. In an *in vivo* study, ER showed a decreased oxidative damage in human retinal pigment epithelial- (ARPE-) 19 cells by Nrf2 and the activation of its downstream shielding phase-II enzymes, namely, heme oxygenase 1 (HO-1) and NADPH, NADPH quinone dehydrogenase 1 (NQO1) [34]. Similarly, ER possesses antitumor effects mainly by modulating ROS in human keratinocyte cells [35]. An in vivo study showed that ER has a role in modulating the signals to the transient receptor potential cation channel subfamily V member 1 or the vanilloid receptor 1 (TRPV1) [36]. TRPV1 is liable for intense and constant involvement in signal transduction and adjustment in changing environment. ER maintains a safe balance in T lymphocytes, natural killer cells, and nitric oxide (NO) concentration which can help improve level of cellular toxicity [37]. In addition, ER significantly diminishes intracellular ROS and lipid peroxidation and restrains normal mitochondrial function and hence saves human hepatocellular disease cells (HepG2) [38]. ER safeguards hydrogen peroxide- (H_2_O_2_-) mediated oxidative damages in prostate cancer (PC12) cells by upregulating the expression of Nrf2/HO-1 and *β*-glutamyl cysteine synthetase pathways [39]. Furthermore, E7G decreases the expression and activation of Nrf2 in cisplatin-induced harmful effects in human mesangial cells [40].

### 3.7. Antidiabetic Potential

ER secured high glucose- (HG-) initiated oxidative stress in glomerular mesangial cells (MCs) mainly by repression of inflammatory cytokines such as tumor necrosis factor *α* (TNF-*α*), interleukin 1*β* (IL-1*β*), and IL-6 levels via reducing Akt/NF-*κ*B pathway which supposes that ER may add to its defensive impacts against HG incitement [41]. A dietary intake of ER in mice decreases lipogenesis-related qualities, and an improvement in insulin resistance was reported by suppressing hepatic gluconeogenesis, enhanced glucose metabolism, and modulated the synthesis and discharge of incretin hormones, namely, gastric inhibitory polypeptide (GIP) and glucagon-like peptide-1 (GLP-1) [42]. The studies suggested that the PI3K/Akt pathway in high-glucose HepG2 and cultured 3T3-L1 adipocytes was improved by ER treatment [28]. These findings suggest that ER can upregulate glucose uptake by improving insulin production. ER is a novel stimulator of insulin production that can be applied for glucose-utilization by means of cAMP/PKA signaling pathway [43]. Moreover, it improves glucose resistance and upgrades plasma insulin in nondiabetic and diabetic rodents. ER specifically reduces the degree of retinal aggravation and plasma lipid peroxidation in early diabetic rodents through downregulation of retinal TNF-*α*, intercellular adhesion molecule 1 (ICAM-1), vascular endothelial growth factor (VEGF), and endothelial nitric oxide synthase (eNOS) and keeps their amount in sub-basal levels [44]. Green tea having extracts of ER potentially destroys cholesterol levels (LDL), supplemented by concealment of 3-hydroxy-3-methylglutaryl-coenzyme A reductase (HMGCR) and 3-hydroxy-3-methylglutaryl-coenzyme A synthase (HMGCS) levels and upregulation of low density lipoprotein (LDL) receptor levels in the liver [45]. Moreover, ER repressed *α*-glucosidase that showed its antidiabetic potential [46].

ER is present in lemon as a natural phytochemical and significantly controls obesity and diabetes [44]. ER has been isolated and characterized as a novel insulin stimulator in both *in vitro* and *in vivo* experiments which could be applied as an elite glucose-suppressant action using cAMP/PKA pathway [43]. In addition, in diabetic rodents that were supplemented with ER, it successfully reduced oxidative stress [7]. The treatment with ER may upregulate the outflow of PPAR*γ*2 and the adipocyte-explicit unsaturated fat restricting protein [44]. Besides, ER exposure fundamentally suppressed diabetes associated lipid peroxidation [47]. ER appeared to provide protection to the rodent retinal ganglion cells- (RGC-) 5 from high glucose-induced oxidative stress and avoided cell apoptosis by means of the activation of Nrf2/HO-1 pathway [48].

### 3.8. Anti-Inflammatory Effects

ER is a characteristic flavonoid that has been accounted for mitigating and being hostile to osteoclastogenic impacts. ER has an impact on provocative reactions in osteoarthritis (OA), where ER lessens the hindrance of cellular functions in IL-1*β*-exposed chondrocytes. Similarly, ER hindered the outflows of inducible nitric oxide synthase (iNOS), cyclooxygenase-2 (COX-2), expression of prostaglandin E2 (PGE2), and NO by activating IL-1*β* production. A huge list of inflammatory cytokines and chain of matrix metalloproteinases (MMPs) were evoked by IL-1*β* treatment that was additionally weakened by ER. Moreover, ER pretreatment repressed the expression of inhibitor of *κ*B*α* (I*κ*B*α*) and p-p65 levels and upregulated the expression of Nrf2 and HO-1 in IL-1*β*-treated chondrocytes. The small interference Nrf2 (siNrf2) treatment essentially restrained the expression of Nrf2 and ultimately its downstream phase II enzymes such as HO-1 in chondrocytes. Furthermore, siNrf2 transfection in a similar way nullified the calming impacts of ER in chondrocytes. This showed that ER has mitigating impacts on IL-1*β*-stimulated chondrocytes, while this impact was interceded by restraining NF-*κ*B by triggering Nrf2/HO-1 antioxidant pathway [49].

ER regulated the levels of the inflammatory cytokines such as TNF-*α*, IL-6, IL-8, and and IL-1*β* signaling and upgraded forkhead box proteins O1 (FOXO1) in RA-FLS in rheumatoid joints pain [50]. FOXO1 is an Akt downstream factor and the latter gets phosphorylated promptly by decreasing levels of the former.

ER appeared to secure the retinal ganglion cells (RGCs) from high glucose-induced oxidative damage and cell apoptosis by initiation of the Nrf2/HO-1 antioxidant pathways [48]. ER cures atopic dermatitis (AD) in mice through inactivation of IL-4 and upregulation of serum immunoglobulin E (IgE) protein [17]. In IL-1*β*-activated chondrocytes, ER eased irritation by repressing NF-*κ*B signaling pathway mainly by activating Nrf2/HO-1 antioxidant pathway [49]. Another outcome indicated that ER improves lipopolysaccharides (LPS)-intervened amyloidogenesis and memory hindrance by restraining the toll like receptor 4, mitogen-initiated protein kinase (MAPK), and PI3K/Akt (phosphatidylinositol 3-kinase/protein kinase B) pathways. On the other hand, ER activates the sirtuin 1 (Sirt1) pathway which impedes the downstream movement of NF-*κ*B signaling cascades [2]. Moreover, ER treatment hindered blood urea nitrogen, creatinine, malondialdehyde (MDA), thiobarbituric corrosive, ROS, TNF-*α*, and IL-1*β* levels of cisplatin-induced stress in renal tissues [51]. ER modulates the stressful effects of macrophages through the phosphorylation of p38 MAPK, extracellular signal regulated kinases 1 and 2 (ERK1/2), and c-Jun N-terminal kinase (JNK) [29]. Moreover, ER secures UV-driven cytotoxicity in keratinocytes by regulating the phosphatase-subordinate keratinocyte signaling pathways p38 MAPK and Akt pathways [29].

### 3.9. Immunomodulatory Effects of ER

Macrophages are a significant component in intrinsic immune system and work essentially as phagocytic cells and pose effects via phagocytosing pathogenic creatures [52]. Likewise, enacted macrophages produce enormous measures for NO from arginine and oxygen by different nitric oxide synthases (NOS) as well as decreasing the inorganic nitrates [53]. NO production in the cells is poisonous to microbes and other human microorganisms. Extreme expression of NO responds to superoxide to give rise to peroxynitrites and later is a potent and toxic oxidant by producing a chain of nitrates which can harm a wide variety of proteins, co-factors, enzymes, and biomolecules (DNA and proteins) in cells. Many natural drugs, metabolic products, and ultraviolet radiations are powerful inducers of macrophage NO creation [54]. Investigation demonstrated that ER hindered NO production by peritoneal macrophages. Additionally, ER downregulated NO development [55]. Additionally, ER diminished the NO generation in LPS-treated Raw 264.7 macrophages [29]. The presence of OH bunches at positions 5, 7, 3′, and 4′ along with the twofold bond in the B ring of ER makes it engaged with the high mitigating impact of flavonoids [56]. The presence of two hydroxyl bunches at the 3′ and 4′ places of the B ring of ER could clarify its mitigating movement. Macrophages complete their vague guard work at the end phase of the phagocytic cycle by initiating the lysosomal phosphatase in the cytosolic vesicles [57]. The movement of lysosomal catalysts like basic phosphatase expanded extraordinarily during inflammation cycle and along these lines caused a great damage to the tissues [58]. The current examination demonstrated that ER diminished lysosomal phosphatase activity, uncovering its curative properties.

### 3.10. Cytoprotective Effects on Kidneys

The defensive impacts of ER on cisplatin- (CP-) induced kidney injury are well illustrated. CP-mediated kidney injury model was built up by using 20 mg/kg of CP. The outcomes indicated that treatment of ER restrained the creation of blood urea nitrogen (BUN), creatinine, MDA, TBARS, ROS, just as the creation of TNF-*α*, and IL-1*β* in kidney tissues initiated by CP. ER additionally upregulated the expression of SOD, CAT, and GSH-PX diminished by CP. Moreover, ER was found to up-control the activation of Nrf2/HO-1 and hinder CP-modulated NF-*κ*B activation in the kidneys. Taking the above together, ER showed a protective role against CP-incited kidney injury mainly by activating Nrf2 antioxidant and repressing NF-*κ*B activation [51].

### 3.11. Hepatoprotective Effects

Liver is an important organ that significantly removes numerous harmful metabolites [59]. The fundamental components of As_2_O_3_-prompted liver injury have not been yet fully demonstrated. Nonetheless, recent studies showed that oxidative stress generated by As_2_O_3_ is the sole cause of liver injury [60–62]. Huge literature reported that anticancer agents had remedial effects against arsenic-induced tissue injuries [63,64]. ER has been accounted for to have anticancer properties [36]; moreover, ER leaves cytoprotective effects on As_2_O_3_-induced liver injury by lessening arsenic mediated obsessive changes in liver tissues. Serum ALT and AST were utilized as biochemical marker of hepatic injury [65]. Moreover, ER hindered As_2_O_3_-initiated ALT and AST creation and these outcomes lead to proposing that ER displayed extensive defensive impacts against As_2_O_3_-instigated liver injury.

Arsenic presentation shows oxidative stress by initiating the creation of ROS in liver tissues [61]. The creation of ROS successfully explained following arsenic exposures. Malondialdehyde (MDA) is a noteworthy lipid peroxidation product that multiplies during oxidative stress [66], and it is suggested to utilize ER to screen its efficacy against oxidative stress [67]. The higher levels of MDA have been reported in the liver cells following arsenic exposure. But the treatment of ER surprisingly diminished As_2_O_3_-generated MDA and ROS production. Besides, the hindrance of SOD, GPX, and CAT upregulation by arsenic was suppressed by ER mainly by scavenging the free radicals and ultimately reducing oxidative stress and liver damage. Nrf2 is an essential leucine zipper protein factor that is accounted for to play a basic role in providing the protection to cancer cells [68]. Activation of Nrf2 prompts the outflow of HO-1, which is a cytoprotective phase-II enzyme for significant heme catabolism [69]. The studies indicated that Nrf2 can be utilized as a successful sub-nuclear factor to neutralize As-mediated harmful effects [70]. Furthermore, ER upregulates the activation of Nrf2 and ultimately HO-1 expression prompted by As_2_O_3_ exposures.

### 3.12. Effect of ER on Lungs

**Figure 2:**
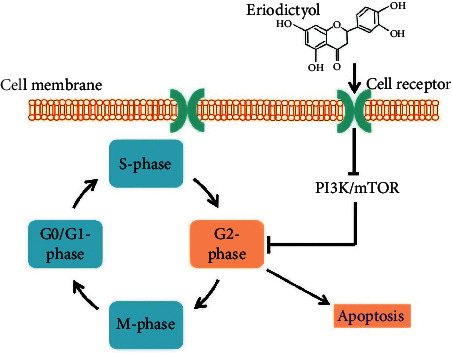
Effect of ER on the cell cycle arrest at G2/M phase, and avoidance of apoptosis. Eriodictyol easily penetrates into the cell via cell receptors and modulates multiple signaling cascades especially PI3K/mTOR that stops the G2-Phase, and hence helps the cells avoid apoptosis.

Lung cancer is one of the deadly cancers around the globe with hundreds of thousands of patients diagnosed annually [71]. Development of drug resistance has made cancer treatment quite difficult. Therefore natural products are known to have multiple targets and are able to suppress or activate complicated signaling pathways to inhibit tumorigenesis [72]. ER showed potential growth-inhibiting activity against human lung A549 cancer cells but least or no toxicity to the normal human lung cells [21]. ER instigates apoptosis in a focused but highly coordinated way. Mitochondrial outer-layer protrusions (MOPs) are a significant cycle engaged with the apoptotic pathway. It is seen that ER decreases the expression of matrix metalloproteinases (MMPs) and shows a fixation subordinate. ER may potentiate apoptosis by combining intracellular decrease of MMPs expression. It has been accounted for that numerous antitumor drugs may target the diseased cells halfway by causing decrease in MMPs expression [73]. Examination of apoptotic cell explained that ER prompts apoptosis and DNA damage in human cellular degradation in the lungs cells [74]. Furthermore, propidium iodide (PI) confirmed the impacts of ER on cell cycle movement, where G2/M cell cycle arrest was seen for a longer time [75]. Also, impacts of ER on Bcl-2/Bax signaling pathway were assessed by immunoblotting that demonstrated that ER pre-treated cells indicated a focused but assisted downregulation of Bcl-2 and upregulation of Bax proteins leading to activate apoptosis (Figure 2). Individuals from the Bcl-2 family proteins, e.g., Bcl-2, Bax, and Bak, are accepted to assume as key controlling factors in the execution of cellular apoptosis, while Bcl-2 family proteins additionally focused to bypass the apoptotic pathway [75,76]. At long last, the impact of ER on the PI3/AKT/mTOR signaling pathway was explored and it was seen that it caused restraint of a key protein of the pathway. These outcomes are fascinating since this objective is viewed as significant malignancy chemotherapy [21].

### 3.13. Neuroprotective Effect of ER

**Figure 3:**
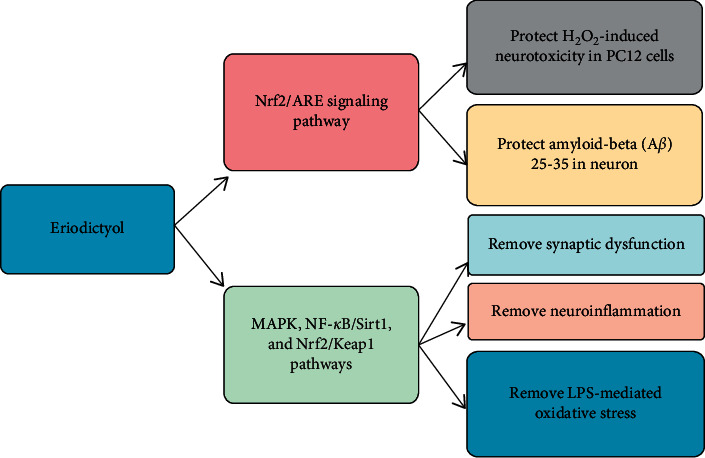
Neuroprotective effects of eriodictyol. Eriodictyol is reported to provide protection via its antioxidant nature by activation of Nrf2/HO-1 cytoprotective pathway. Moreover, Nrf2/HO-1 pathway protects H_2_O_2_-induced neurotoxicity in prostate cancer cells (PC12), and amyloid-*β* in neurons, while having anti-inflammatory effects involving MAPK, NF-*κ*B pathway.

ER has been accounted for to have neuroprotective impacts by activating the cellular cancer prevention agents that may protect the cells (Figure 3). It has been discovered that ER secures hydrogen peroxide- (H_2_O_2_-) instigated neurotoxicity in PC12 cells by means of activation of the Nrf2/ARE cancer prevention agent pathways [39]. Thus, amyloid-*β* (A*β*) 25-35-activated cell demise in primary cultured neurons is incompletely reduced by ER treatment by means of initiation of the Nrf2/ARE signaling pathway [77]. Another *in vivo* and *in vitro* study exhibited that it eases LPS-modulated oxidative stress, neuroinflammation, and synaptic brokenness through the activation of MAPKs, NF-*κ*B/Sirt1, and Nrf2/Keap1 pathways [2]. Here, MAPKs fundamentally control incendiary reactions constrained by NF-*κ*B. Moreover, ER indicated the promising impact of neuroprotection in mice through the guideline of myeloperoxidase, inducible nitric oxide synthase (iNOS), TNF-*α*, and glial fibrillary acidic protein [78]. The Nrf2/ARE pathway assumes a significant part in the recruitment of phase-II detoxifying compounds and cell reinforcement proteins. In addition, E7G was uncovered to critical insurance against cerebral ischemic injury through the upregulation of the Nrf2/ARE pathways [31].

### 3.14. Analgesic Activity of ER

The transient receptor potential (TRP) family speaks to a superfamily of particle channels shaped by six transmembrane spaces that are equipped for penetrating cations, fundamentally calcium. These TRPV1 channels are being expressed in every human cell type of all tissues and are linked for the regulation and modulation of multiple cellular functions in different diseases [79,80]; in particular, TRPV1 receptors have great roles in pain treatment [81]. To discover new components that connect with this receptor, the flavonoid ER was tried. ER repressed the known modulator to the TRPV1 receptor and restrained the Ca^2+^ interceded by capsaicin. ER likewise had an antinociceptive impact in the intraplantar and intrathecal capsaicin tests and being antihyperalgesic and hostile to allodynic impacts in the CFA test. Moreover, it saw that ER completely forestalled the oxidative stress instigated by capsaicin in the spinal line without modifying driving movement or internal heat level [36]. With regard to new compounds that may collaborate with the TRPV1 receptor, we zeroed in on mixes found in therapeutic plants with known antinociceptive impacts. Certain plant extracts in [3H]-RTX needs to explore in detail to figure out which particular active compounds potentially targets and modulates vanilloid site of TRPV1. In view of recent studies, ER dislodges [3H]-RTX officially with more noteworthy strength (around 47 nM) than the old style TRPV1 agonist capsaicin (roughly 3200 nM) [82]. A capsaicin-intervened calcium influx was measured to decide if ER restricting prompts practical balance of the TRPV1 receptor. ER hindered Ca^2+^ inundations with power like that seen in the coupling test and had no impact on Ca^2+^ flooding without capsaicin. This shows that ER goes about as a rival of the TRPV1 receptor and that it restrains Ca^2+^ overflow with more noteworthy power than some old style TRPV1 opponents [83], 5-iodoresineferatoxin (56.7 nM) [84] and capsazepine (7.7 *μ*M) [85].

### 3.15. Antipyretic and Antinociceptive Activities

**Figure 4:**
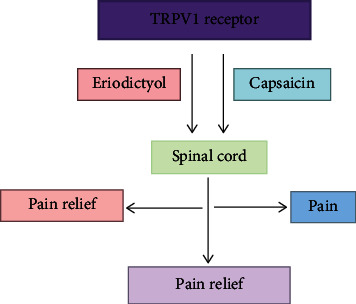
Effect of eriodictyol on pain relief. Eriodictyol mainly hits TRPV1 receptor-mediated activation of the spinal cord and regulates it to relieve pains.

The TRPV1 receptor is the most encouraging objective for the improvement of new pain relieving drugs [86]. The vanilloid receptor is appropriated both in the fringe, where it goes about as a sensor of poisonous upgrades, and in the spinal cord, where it partakes in the transmission of torment [87]. Subsequent to confirming that ER goes about as an enemy of the TRPV1 receptor *in vitro*, our following stage was to decide if it could weaken the nociception incited by capsaicin. ER had antinociceptive impacts with high adequacy and intensity when managed either orally or intrathecally. Besides, oral ER administration constricted the nociception activated by intrathecal capsaicin, which proposed that orally regulated ER may arrive at the spinal cord. Hyperthermia is a typical result of TRPV1 rivals, and it was found that ER had no impact on internal heat level in AMG9810-mediated hyperthermia in mice. Organization of AMG517, a simple form of AMG9810, as of late appeared to cause checked and persevering hyperthermia (≈40°C) in people at portions less than those important to evoke a pain-relieving impact (Figure 4). The AMG517-generated hyperthermia has been discovered to be impervious to traditional medicines for fever, e.g., acetaminophen. This hyperthermia was credited to an expansion in fringe vasoconstriction, which diminishes heat misfortune through the skin, expanding the internal heat level [88]. ER has appeared to cause vasorelaxation [89], which could clarify how ER reduces actuate hyperthermia. This information proposed that ER might be a more desirable treatment than many others for the currently known TRPV1 rivals.

Studies showed that capsaicin-incited nociception happens in association with spinal oxidative pressure. Treatment with NAC, a ROS forager, weakened both the oxidative pressure and the nociception prompted by capsaicin. Accordingly, ROS creation has all the earmarks of being a significant instrument of agony acceptance/transmission in mice [90]. The TRPV1 receptor appears to assume a basic part in oxidative stress intervened nociception. The incitement of TRPV1 receptors may instigate oxidative worry in tangible neurons and the spinal rope, and a few oxidants may adjust TRPV1 receptor work [90–92]. Here, we found that ER forestalled the advancement of oxidative worry in the lumbar spinal string. This impact might be expected either to coordinate opposition of the TRPV1 receptor or to have a cancer prevention agent impact. To recognize these other options, we played out the ABTS test and affirmed that ER likewise has a cancer prevention agent impact [92]. Despite the fact that ER has a roughly 38-overlay more noteworthy intensity for threat of the TRPV1 receptor than for cancer prevention agent action, an immediate cell reinforcement impact could contribute, in any event to a limited extent, to its antinociceptive impact [36].

### 3.16. Miscellaneous Activities

ER could prevent and cure osteolytic diseases characterized by the presence of abnormal osteoclast development mainly due to activity of receptor activator of nuclear factor kappa-B ligand (RANKL) that triggers the differentiation in morphology and functions of osteoclasts via activation of multiple signaling pathways, specifically NF-*κ*B, MAPKs, and Ca^2+^-dependent signaling, which ultimately activates critical transcriptional factor nuclear factor of activated T-cell cytoplasmic 1 (NFATc1).

Furthermore, it hinders c-Fos and NFATc1 expression, with reduced activation of osteoclast specific genes, namely, cathepsin K, Vacuolar-type H + -ATPase d2 subunit, and tartrate-resistant acid phosphatases (TRAcP/Acp5) [93]. In continuation to this, it is also linked with the avoidance and proper treatment of osteolytic harms identified with multidirectional deviantly expanded expanding osteoclast advancements and capacity. Ribosomal S6 kinase-2 (RSK2) and activating transcription factor 1 (ATF1) play a critical role in transforming neoplastic cells and ER suppressed RSK2 kinase activity in epidermal growth factor- (EGF-) mediated neoplastic cell transformations [94].

Recently, an in silico molecular docking study reported that ER has good binding energies and antiviral effects by its cellular multi-target ability against multiple proteins from SARS-CoV-2, especially the angiotensin converting enzyme 2 (ACE2) [95]. Moreover, compared to many already known drugs, ER strongly binds to virus replicating proteins such as SARS-CoV-2 helicase which potentially helps recognize RNA: DNA duplex is a unique feature of viruses, and active sites of SARS-CoV-2 spike glycoprotein C chain having 10 amino acids [95]. Hence, ER appeared to be a novel multi-target molecule for repurposing against SARS-CoV-2.

## 4. Conclusions

All the flavonoids are quite important for therapeutic effects, but ER is the best one for prevention and treating various ailments. ER is extracted from natural sources from various fruits and hence no toxicity or side effects are reported for its use. ER alters the cellular biochemistry and molecular mechanisms for the prevention of onset of multiple diseases. In-depth studies will offer new insights of knowledge and surely will add to another era of flavonoid based therapeutics and nutraceutical options for the treatment of oxidative stress driven diseases.

## Data Availability

All data used to support the findings of this study are included within the paper.
